# Identify structures underlying out-of-equilibrium reaction networks with random graph analysis†

**DOI:** 10.1039/d4sc05234j

**Published:** 2025-01-08

**Authors:** Éverton F. da Cunha, Yanna J. Kraakman, Dmitrii V. Kriukov, Thomas van Poppel, Clara Stegehuis, Albert S. Y. Wong

**Affiliations:** a Department of Molecules and Materials, Faculty of Science and Technology, University of Twente Drienerlolaan 5 Enschede 7522 NH The Netherlands; b MESA+ Institute and BRAINS (Center for Brain-inspired Nano Systems), University of Twente Drienerlolaan 5 Enschede 7522 NH The Netherlands; c Department of Mathematical Operation Research, Faculty of Electrical Engineering, Mathematics and Computer Science, University of Twente Drienerlolaan 5 Enschede 7522 NH The Netherlands c.stegehuis@utwente.nl

## Abstract

Network measures have proven very successful in identifying structural patterns in complex systems (*e.g.*, a living cell, a neural network, the Internet). How such measures can be applied to understand the rational and experimental design of chemical reaction networks (CRNs) is unknown. Here, we develop a procedure to model CRNs as a mathematical graph on which network measures and a random graph analysis can be applied. We used an enzymatic CRN (for which a mass-action model was previously developed) to show that the procedure provides insights into its network structure and properties. Temporal analyses, in particular, revealed when feedback interactions emerge in such a network, indicating that CRNs comprise various reactions that are being added and removed over time. We envision that the procedure, including the temporal network analysis method, could be broadly applied in chemistry to characterize the network properties of many other CRNs, promising data-driven analysis of future molecular systems of ever greater complexity.

## Abstract 2

Temporal network analysis reveal when feedback interactions emerge in an out-of-equilibrium reaction network. This method could be broadly applied to provide data-driven analysis of experimentally-designed chemical reaction networks.

## Graphical abstract

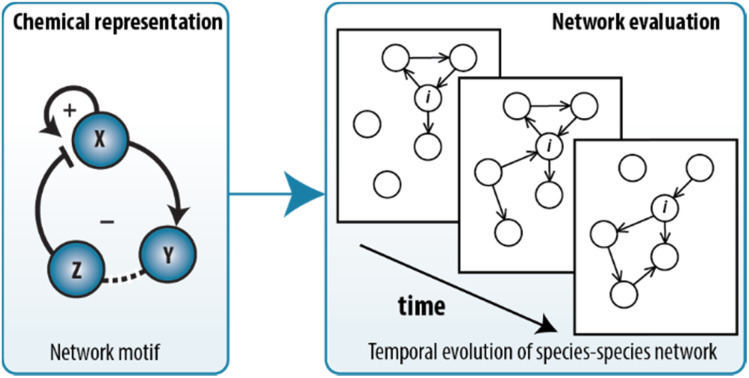

## Introduction

Complex networks describe the interactions between a vast number of components and are the foundation of many natural, societal, and technological phenomena.^1^ At the molecular level, interactions take place in networks based on DNA, proteins, or metabolites.^2^ The discovery of recurrent patterns in biochemical networks (*i.e.*, network motifs),^3^ in particular, has sparked chemists to translate theoretical design principles that use feedback loops^4,5^ into practical molecular methods that enable the synthesis of out-of-equilibrium chemical reaction networks (CRNs).^6,7^ Despite significant progress, expanding the complexity in CRNs in a modular, theory-based way remains scarce.^8^

One of the main difficulties in the synthesis of CRNs resides in the requirement of the integral approach, involving organic synthesis and mathematical modelling.^9^ Advances in systems chemistry—a subdiscipline at the interface of systems biology and supramolecular chemistry^8^—demonstrate that CRNs can be experimentally designed, rationalizing behaviour ubiquitous for living systems *e.g.*, bistability,^10–12^ oscillations,^13,14^ and other transient behaviours.^15–17^ In all examples, mass-action models^18^ are used to support and validate their observed dynamics, demonstrating the need for mathematical modelling to guide the design of CRNs.^19^ Experimentally-designed CRNs (or, more generally, CRNs designed artificially by chemists), however, cannot always be accurately modelled using mass-action kinetics due to complexity of the reaction mechanisms involved as well as limitations in the determination of rate constants thereof.^20^ The experimental framework for CRNs with more complex behaviour^21–23^ is gaining ground rapidly, and novel approaches that can capture their dynamics are needed.

Alternatively, mass-action models can be represented by a bipartite graph with nodes representing chemical species and reactions. The mathematical foundation for so-called species–reaction graphs was introduced in the early 1970’s,^24^ and gained significant attention after Feinberg demonstrated that abstract graph theory can be applied to study interconnected chemical reactions.^25,26^ Bipartite graphs are commonly applied to represent CRNs,^27,28^ or to detect the lowest-energy paths in molecular transformations (wherein reaction mechanisms are treated as CRNs).^29–31^ In all examples, paths are used as network properties. Other network statistics (such as centrality, clustering and motifs) have been used to analyse CRNs that are large. In this context, biochemical networks^32–34^ and combustion networks^35^ are similar because they both comprise many intermediates, each with numerous reaction paths. When analysing network statistics, random graphs can provide a benchmark to filter out effects that are created by randomness. Overall, mathematical properties of the solutions of mass-action systems are strongly related to key properties of the CRNs that generate them, including their feedback interactions.^36^

Despite the advances in CRN theory, analyses of species–reaction graphs that are sufficiently informative to guide the design of CRNs for the synthesis-oriented chemist are scarce. An important distinction between the out-of-equilibrium reaction networks of interest to the systems chemist^8–17^ and the aforementioned networks, wherein reaction mechanisms are treated as CRNs,^27–31,35^ is that the first type of CRNs use intermediates that are stable and separable compounds whereas the second type of CRNs use intermediates that are short lived and cannot be separated as individual compounds. That is, while theory can take the perspective that thousands of reactions can be treated as similar, in practice, each class of reactions can have its own chemical rules.^37,38^ We need new methods to represent, collect and extract information to predict structures and functions of CRNs that are synthetically feasible. This is a non-trivial task as the design of CRNs requires expertise in chemical synthesis and graph analysis to create a foundation that takes into account the chemical and mathematical perspectives on how reactions become networks.

**Fig. 1:**
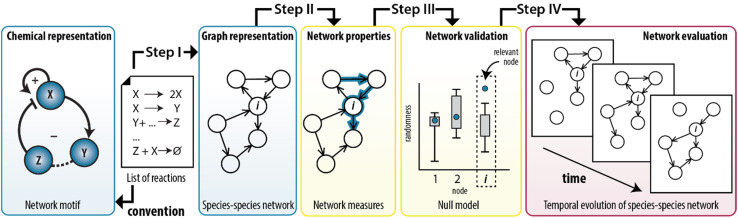
Translation of synthetically-designed CRNs into a species–species network enables graph-based analyses. A list of reactions can be visualized as a conceptual model comprising feedback loops (left panel, conventional approach) and as a species–species network (step I). In this work, such representation is used to simulate the structure of the CRN by applying network measures (step II) and assessing their significance (step III). The procedure can also take the dynamic nature of chemical reactions into account (step IV).

In this work, we show that we can combine the temporal dynamics of the CRN with the information provided by the graph to analyse the temporal behaviour of the network statistics. We developed a method that integrates the representation of CRNs as graphs with the application of network measures to model experimentally-designed CRNs (Fig. 1). Briefly, any chemical system can be described by a set of elementary reactions but to create insights into the system, chemists typically use conceptual models to visualize the interactions as positive and a negative feedback loops (Fig. 1, left panel). To illustrate, ESI, Fig. S1† shows a previously developed enzymatically-driven CRN for which a mass-action model was developed.^13^ Building on this foundation, we designed a procedure (illustrated in Fig. 1 as steps I–IV) that could translate the reactions in this model into a species–species network comprising of nodes and edges. This procedure not only allows for an explicit illustration of the relationships among the nodes, but also for the application of network measures commonly employed in network science. We show that measures for clustering and centrality could reveal the underlying network statistics important for the temporal existence of feedback loops. To the best of our knowledge, this is the first work that combines random graph theory with dynamics of out-of-equilibrium reaction networks.

## Results and discussion

### From elementary reactions to structure (step I)

The first step in the procedure encodes the enzymatic oscillator as a species–species network (ESI, S2†). In here, each edge represents a unique chemical conversion from one species to another represented by nodes. Our procedure transforms the set of elementary reactions into a network. The species are labeled as reactants, intermediates and products resulting in a total of 10 nodes; three reactants (Tg, Ap, Pro-I), six intermediates (Tr, Int-I, I, Tr·Tg, Tr·Pro-I, Ap·Int-I), and one product (P) that summarizes the species that do not contribute to the overall dynamics (the inhibited trypsin complex and hydrolysed inhibitors). Subsequently, we assigned a directed edge for each pair of species in an elementary reaction, with the direction of the edge indicating whether the species was consumed or produced. We note that multiple chemical conversions could take place between the same pair of species.^39^ To account for this multiplicity, we defined weights based on the number of edges assigned to each pair of species. ESI, Table S1† show the outputs of the algorithm applied on the enzymatic oscillator, where the final column (a so-called edge list) defines the species–species network: a graph with 10 nodes and 19 weighted edges (G_1_).

### Network properties of the enzymatic oscillator (step II)

**Fig. 2:**
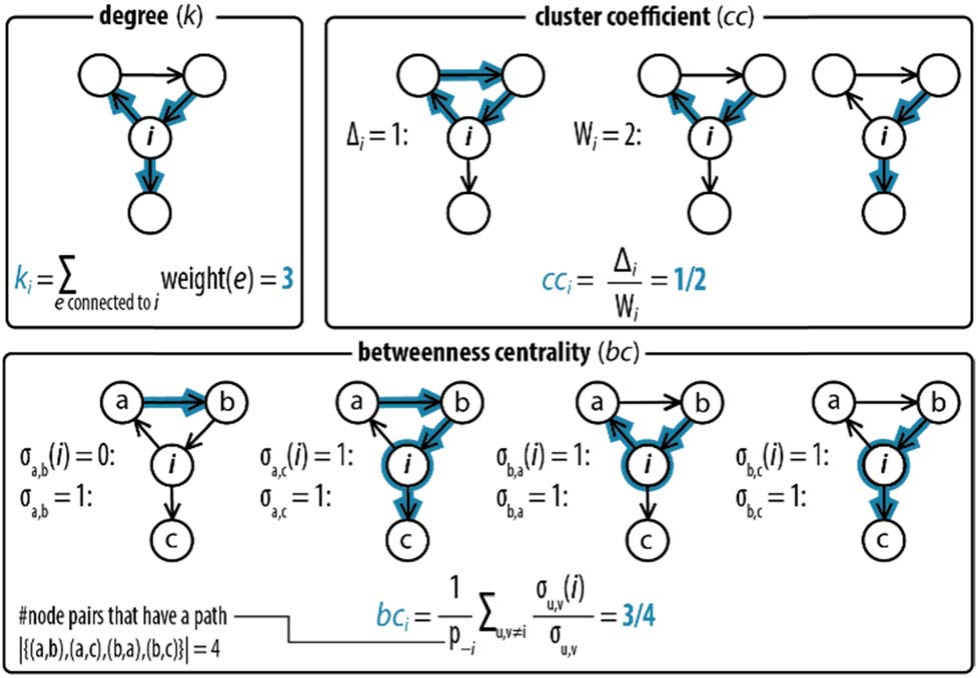
Degree, clustering coefficient and betweenness centrality. An example for how network measures are determined for node *i*, where all edges have weights equal to 1. The degree (*k*_*i*_) measures the sum of the weights of the connections of each node. The clustering coefficient (cc_*i*_) measures the likelihood that two neighbours of a node forming a wedge form a feedback triangle (*Δ*_*i*_). A wedge, *W*_*i*_, is a set of two edges *h* → *i* and *i* → *j* with *h* ≠ *j* at node *i*. A feedback triangle (*Δ*_*i*_), therefore, is *h* → *i* → *j* → *h*. The betweenness centrality measures how frequent a node lies on the path with the least number of steps between other nodes. It is computed by counting the number of shortest paths that pass through node *i*.

Next, step II in the procedure is to choose and adapt the network measure of interest. To illustrate, we chose three network measures^2,40^ (degree, clustering coefficient, and betweenness centrality, Fig. 2) commonly applied in network science. To account for the directed and weighted nature of the edges in our species–species network, we adapted these measures slightly.^41,42^ To be precise, the first measure, degree (*k*_*i*_), computes the sum of the weights of the edges connected to each node:*k*_*i*_ = ∑_*e* connectedto *i*_weight(*e*)In other words, *k*_*i*_ determines the number of times that a node is involved in the species–species network.

The second measure, clustering coefficient (cc_*i*_), investigates paths that form cycles between triples of nodes *i*, *j* and *k*. Such paths are called ‘feedback triangles’, and they are of the form *i* → *j* → *k* →*i*. The clustering coefficient computes the fraction of feedback triangles formed by the node with its neighbours. In particular,
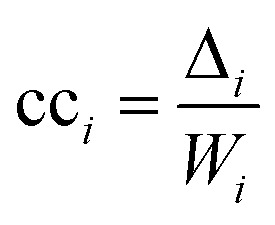
where *Δ*_*i*_ denotes the number of feedback triangles attached to node *i*, and *W*_*i*_ denotes the number of wedges, the number of length two paths passing through node *i*. The cc_*i*_ measures, thus, the likelihood that two neighbours of a node forming a wedge can form a feedback triangle. For instance, cc_*i*_ = 1/2 in Fig. 2 means that half of the wedges attached to node *i* form a feedback triangle. Note that this definition of cc_*i*_ does not consider edge weights.

The third measure, betweenness centrality (bc_*i*_), computes the centrality of a node based on the shortest paths in the network: for every pair of nodes, node *i* may lie on the path with the least number of steps between these nodes. The bc_*i*_ outputs the fraction of nodes for which this is true. In particular,
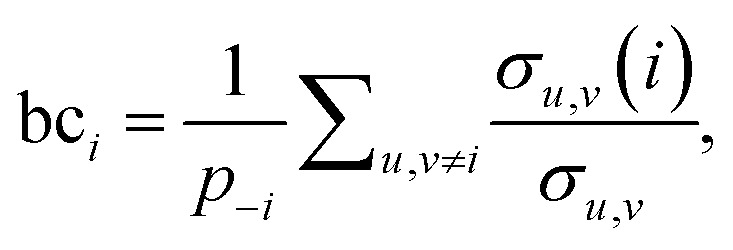
where *p*_−*i*_ is the number of node pairs (excluding node *i*) between which a path exists, *σ*_*u*,*v*_ is the number of shortest paths between nodes *u* and *v*, and *σ*_*u*,*v*_(*i*) the number of shortest paths between *u* and *v* that pass through node *i*. The multiplicity of a shortest path between two nodes is defined as the product of the edge weights of the path. bc_*i*_, thus, provides information on the number of times with which a species acts as an intermediate between network connections.

**Fig. 3:**
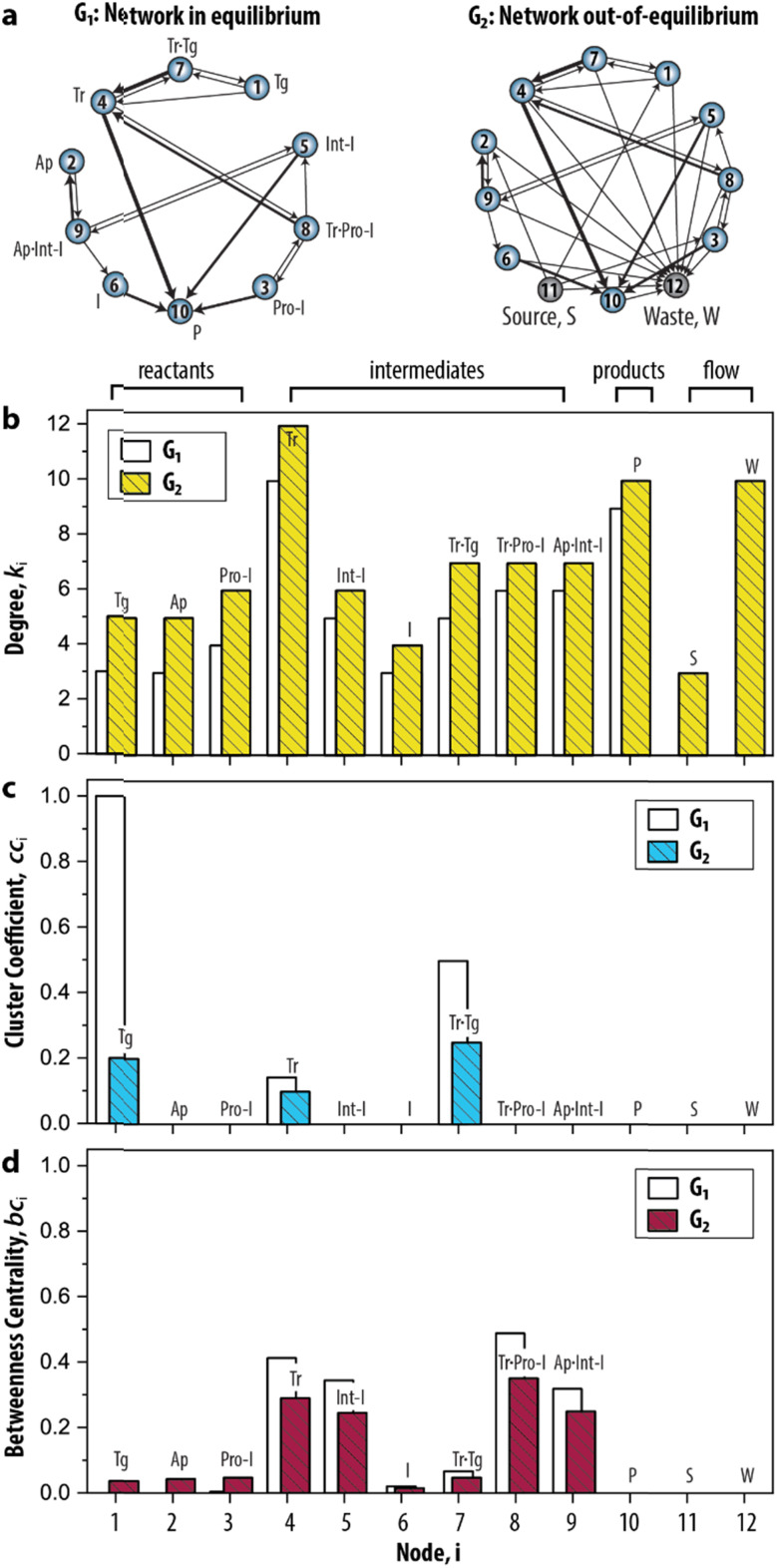
Static analysis of the species–species network of the enzymatic oscillator using network measures. (a) Graph-based models, or species–species networks, of the enzymatic oscillator in- and out-of-equilibrium. G, abbreviates graph. Source, S, is the node that accounts for the inflow of the reactants, and waste, W, is the node that accounts for the outflow of the products of the network. (b–d) Network measures applied on the enzymatic oscillator. Data are reported in ESI, Table S3.†

Having established the foundation for the three network measures, we then applied the measures to characterize the species–species network (ESI, S3.1†). Crucially, the oscillator can only exist when the network is maintained under out-of-equilibrium conditions. Therefore, we introduced two nodes (source, S, and waste, W) to take the inflow of the reactants and the outflow of the products of the network into account. Fig. 3a shows that the graph for the CRN out-of-equilibrium (G_2_) comprises more nodes (12 instead of 10) and more weighted edges (32 instead of 19) than the graph for the CRN in equilibrium (G_1_). Details of the graphs are appended to ESI, Tables S1 and S2.†

In this CRN, the intermediates are nodes that on average have a higher contribution than other species to the connectivities in the graph, given that their degree (*k̄*_4–9_ = 7.1) is higher in comparison to the degree of the reactants (*k̄*_1–3_ = 5.3) (Fig. 3b). The node 10 has an artificially high value (*k*_10_ = 10) because it summarizes several species as products. The degree for nodes 11 and 12 that represent the flow components, *S* (*k*_11_ = 3) and *W* (*k*_12_ = 10), reflects the inflow of the three reactants and outflow of ten species. That the node for Tr (*i* = 4) has the highest degree (*k*_4_ = 12) reflects the fact that the design of the CRN is built around the key enzyme Tr (see conceptual model in ESI, Fig. S1†).^13^

A different pattern emerges when we considered the cc_*i*_ (Fig. 3c). On the node for Tg (*i* = 1), for instance, cc_1_ = 0.2 as 1/5 of the wedges can create the feedback triangle representing the conversion of Tg into Tr (*via* the auto-activation, Tg → Tr, and autocatalytic steps, Tg + Tr = Tr·Tg → 2 Tr). For Tr·Tg, node 7, cc_7_ = 1/4 is slightly higher because it has the same number of triangles but one less wedge. The cc_*i*_ for 4, species Tr, is significantly lower (cc_4_ = 1/10) as this node has neighbours that are not shared with 1 and 7. Overall, we observed two distinct responses: a cc_*i*_ for any node is (i) non-zero for species that are involved in the positive feedback loop (Tg, Tr, and Tr·Tg), and (ii) zero for species that are not involved in the positive feedback loop. Notably, the auto-activation step was included as a side reaction in the mass-action model of the enzymatic oscillator as it was required to provide the initial activation of Tr.^13^ In this case, the auto-activation, plays a critical role as well, because it is required to complete the feedback triangle between 1, 4 and 7, making the numerator of cc_*i*_ non-zero.

The bc_*i*_ (Fig. 3d) shows that nodes 4 and 5 have the highest betweenness centrality value. This underscores the importance of the species Tr and Int-I to act as the key intermediates of the network. Surprisingly, the nodes 8, and 9—nodes for species which role in the oscillator is often neglected in the mass-action model, Tr·Pro-I and Ap·Int-I—also appear to share a similar high occurrence with which the species act as an intermediate between network connections. In contrast, species *I* (bc_6_ = 0.05) appears to have a less important role to sustain the network connectivity than anticipated in previous work.^13^ Other nodes with low values for bc_*i*_ are 1–3, and 7. The nodes for the reactants (*i* = 1–3) only appear on shortest paths that direct from an inflow (S). The node for Tr·Tg (*i* = 7) only lies on the shortest path corresponding to when Tg converts into Tr, explaining its similarly low betweenness value. Finally, the three nodes, *i* = 10–12, do not lie on any shortest path. bc_10_ is zero and validates that 10 summarizes species that do not contribute to the overall dynamics. bc_11_ and bc_12_ are zero is because edges are either directing from 11 (the source) or directing to 12 (the waste). Overall, that similar patterns for *k*_*i*_, cc_*i*_, and bc_*i*_ were observed for G_1_ shows that the introduction of the nodes to account for the continuous inflow of reactants and outflow of products does not significantly alter the structural properties of the CRN.

### The significance of network measures applied on the enzymatic oscillator (step III)

In step III of the procedure, the significance of the network measures is assessed by a random graph null model. This is important, as often the measured values depend, in unknown ways, on the CRN size, its edge density and other intrinsic system properties.^43^ For this reason, it is common in network science to compare statistics on a given network to randomized versions of the same network, to see whether they stand out, *i.e.*, are significant.^43^ This approach has proven extremely successful in *e.g.*, metabolic, genetic and protein networks,^2^ and we propose it can be applied to out-of-equilibrium networks relevant to systems chemistry. We developed a random graph null model to compare the statistics of G_2_ to randomized versions of the same species–species network. The procedure generates 10 000 randomized configurations of G_2_, under the condition that the degrees are fixed but connections are randomized. Subsequently, the cc_*i*_ and bc_*i*_ are applied to these randomized networks to determine the range of values that these measures can take (ESI, S3.2†). In other words, we provide a negative control for the measures applied on G_2_ (a so-called null hypothesis). In this way, we can identify which nodes show features that may be used to characterize the trypsin oscillator.

**Fig. 4:**
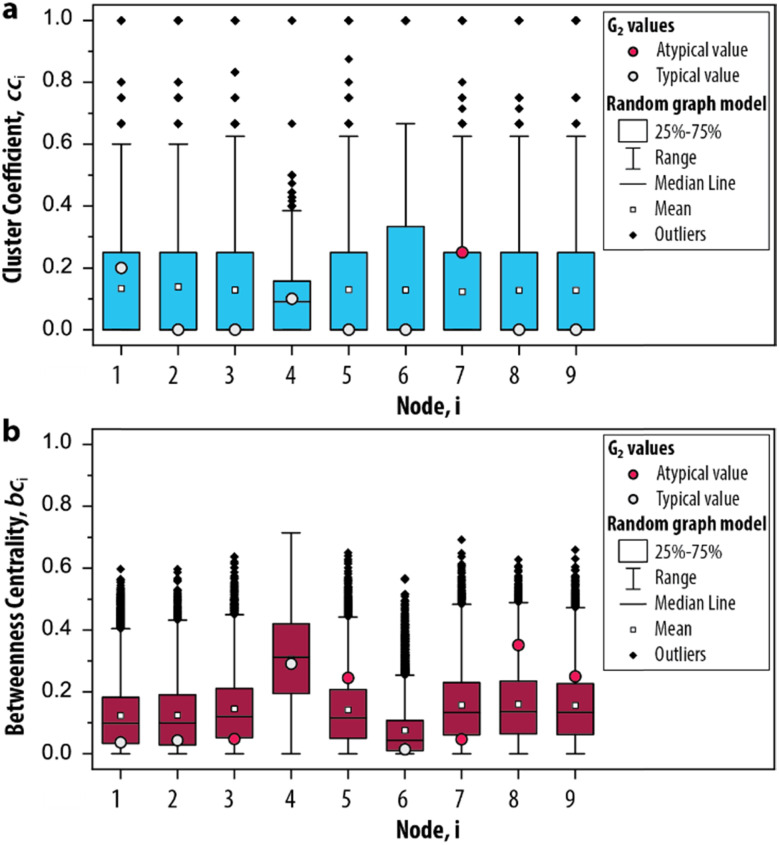
Significance of the network measures. Box plots depict the distribution of the measures based on 10 000 randomized graph samples for the (a) clustering coefficient and (b) betweenness centrality. The relevant nodes for G_2_ are determined by whether the value from the original network occurs within the 25–75th percentile (*i.e.*, a typical value) or not (*i.e.*, an atypical value).

The possible values that the measures can take, given random conditions, are summarized as boxplots in Fig. 4a and b. The distributions for cc_*i*_ and bc_*i*_ based on the null model are compared with the values obtained for G_2_ to determine if its node occurs within the 25–75th percentile of the randomized networks (*i.e.*, a typical value, depicted as a grey circle). A typical value indicates that the properties of the node would also appear if the CRN was driven by random processes. On the other hand, a node that is not within the 25–75th percentile (*i.e.*, an atypical value, depicted as a red circle) indicates that their properties are different from an otherwise randomly assembled CRN. For the clustering coefficient, we found that only 7 appears to be atypical (Fig. 4a) and for the betweenness centrality, 3, 5, 7, 8 and 9 all appear atypical (Fig. 4b). The nodes 10–12 are excluded from this analysis as they did not contribute to the overall dynamics of the system. Hence, we identified the relevant set of nodes for the graph-based analysis of the enzymatic oscillator under out-of-equilibrium conditions. That is, for further analysis of the cluster coefficient there is only one relevant node (7) and for the betweenness centrality there are five nodes from which we can choose (3, 5, 7, 8 and 9).

### Network measures applied on temporal behaviour (step IV)

**Fig. 5:**
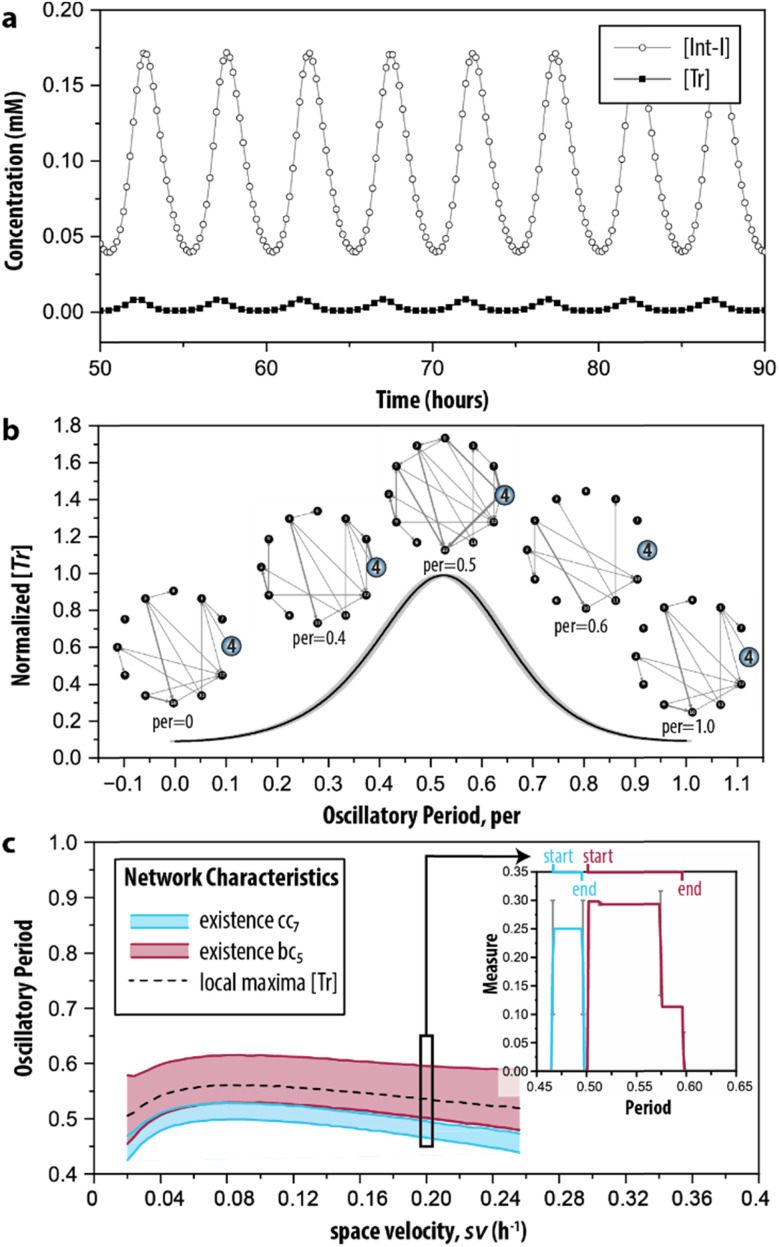
Time-dependent graph-based analysis. (a) Simulated time series of the enzymatic CRN wherein oscillations of key intermediates (Tr and Int-I) are sustained. See ESI, S3† for initial conditions. (b) The evolution of network G_2_ during the time required for completing a single oscillation (oscillatory period). The normalized [Tr] is determined by averaging five consecutive oscillations in (a). (c) The emergence of the cluster coefficient (cc_*i*_) and betweenness centrality (bc_*i*_) as a function of space velocity (sv), the parameter that determines the inflow and outflow of species. The insert shows the appearance of cc_7_ and bc_5_ during the oscillatory period under the conditions depicted in (b) (sv = 0.2 h^−1^). Extended materials include animated image sequence used in (b), and (c).

Finally, in step IV of the procedure, the time-dependent dynamics of the network measures is analysed. This is done, since reactions take place all the time but at specific moments during the reaction trajectory certain ones are more prevalent (depending on the change in the concentration of the species involved in the reaction). Therefore, we simulated the CRN using the model described by ODEs. Fig. 5a shows the characteristic behaviour of the oscillator in time, wherein the concentration of key intermediates Tr, [Tr], and Int-I, [Int-I], oscillate with the same frequency. The simulated time series are used as input to represent the temporal evolution of the species to determine the addition or removal of edges in G_2_. Briefly, we considered that an outgoing edge can only exist when the associated node is present, and used a threshold to determine the presence of the nodes at each time interval (for details, see ESI S3.2†). To ensure that the sustained oscillations had established, our analysis starts from 50 h onwards.

During the development of each oscillation, different subgraphs of G_2_ were identified. Fig. 5b shows the behaviour of the CRN as a single oscillation with the oscillatory period ranging from 0 to 1. The inserts highlight the node for Tr (*i* = 4). At the local minima of the oscillation (per = 0 and per = 1) the node is only connected with one edge in the graph, whereas at the local maxima (per = 0.5), the node is connected with many edges. When transitioning from a minimum to a maximum (*e.g.*, per = 0.4), edges are formed, but they disappear during the transition from a maximum to a minimum. Particularly, at per = 0.6 the node becomes disconnected from the graph. The extended set of ‘snapshots’ is appended to ESI, Fig. S4,† which shows that the discussed transitions are rather abrupt and that, remarkably, new edges are often being formed, even during per between 0.5 and 1. Hence, the network with all chemical interactions involved does not exist, and moreover, while the trend in the total number of reactions that are active may go up or down, the system still constantly adds and removes individual reactions.

The graph-based analysis was extended by determining when the clustering coefficient of 7 (cc_7_) and betweenness centrality of 5 (bc_5_) are non-zero during the oscillatory period. The cc_*i*_ and bc_*i*_ of the discussed example are indicated in the insert of Fig. 5c, which shows that cc_7_ could yield its maximal value of 1/4 only within per = 0.47–0.49 (thus, before the local maximum of the oscillation). The maximal value of 0.30 for bc_5_, instead, was found at a different interval, 0.5–0.58 (thus, when the oscillation started to decrease). We note that 7 was the only option for the temporal analysis of cc_*i*_, and that 5 was chosen arbitrarily for the temporal analysis of bc_*i*_. The latter can be substituted by any of the other relevant nodes identified in the random null model (3, 7, 8 and 9) without influencing the analysis.

Essentially, the clustering coefficient and betweenness centrality show if (and when) the desired feedback loops are acting on the network. Fig. 5c shows the impact of different initial conditions on network dynamics, measured by the appearance of the network measures cc_*i*_ and bc_*i*_ under a range of space velocities, sv—the parameter that determines the rate of inflow and outflow of species. The cc_*i*_ and bc_*i*_ cannot distinguish oscillations that are sustained or damped (ESI, Fig. S5†). To this end, we determined the intervals for their presence only for sv values within 0.02 h^−1^ ≤ sv ≤ 0.25 h^−1^, the conditions wherein oscillations are sustained. We found that the trajectories of cc_7_ and bc_5_ follow the pattern of the local maxima of the oscillation in [Tr] (dashed line). Fig. 5c shows the (dis)appearance of the connection with the intermediate inhibitor (*i* = 5), highlighting the emergence of the reactions important for a short, or efficient, pathway in G_2_. In this case, the edges that are present when bc_5_ reached its maximum value indicate that the connections crucial for the negative feedback loop are made. Therefore, bc_5_ appears when the oscillation decreases. Oppositely, the clustering coefficient allows for examining the existence of the positive feedback. In the case of this CRN, it was built on three species forming a feedback loop, and Fig. 5c indicates that it is present only in a narrow interval.

## Conclusions

Graph-based analyses are widely used in literature for basic characterization of networks of any kind but have rarely been applied to experimentally-designed chemical reaction networks (CRNs). We have developed a method to characterize experimentally-designed CRNs, which we demonstrated based on an existing enzymatic network. The method comprises four steps: (I) represent a CRN as a graph to model the interactions as edges. (II) Apply network measures to provide the key characteristics of the structure underlying the CRN. (III) Identify the crucial nodes in the network using a random graph null model. (IV) Perform temporal analysis on the graph to reveal the evolution of the structure over time. This method allows us to analyse systems with out-of-equilibrium behaviour such as oscillations in which the standard CRN theory of does not apply. The integration of the expertises in system chemistry and random graph analysis ensured that the translation from a reaction scheme that defines the CRN into a graph as well as the opposite translation—from network measures into the chemical functioning of the CRN—was executable.

The algorithm developed in this work can be used for translating many other synthetically-designed CRNs into species–species networks, providing ample opportunities to extend the analysis beyond the clustering coefficient and betweenness centrality and apply it to a larger set of out-of-equilibrium networks. Most of the current measures are built on the occurrence of specific network patterns of specified sizes,^43^ while in systems chemistry, similar structures such as feedback loops may result from differently sized network patterns. Characterizing CRNs with more complex feedback loops will require network measures capable of including longer and more diverse cycles. Furthermore, this may require constructing these models on more complex graph-like structures, such as hypergraphs,^44,45^ where it becomes possible to identify higher-order paths (known as hyperpaths). These hyperpaths provide a more detailed view of the feedback loops in complex interactions, capturing the intricate dependencies within the networked chemical system. Testing newly designed measures would require a broader training set of CRNs to assess their validity, underlining the need for developing such a database.^46^ We anticipate that our approach forms a foundation that may lead to training algorithms that can go beyond the synthesis of complex molecules^47,48^ and enable data-driven analysis of complex out-of-equilibrium reaction networks with ever greater complexity.

## Methods

### Software

NetworkX (an open source Python package for complex networks) was used as the computational data structure to develop the species–species networks and implement the network measures used in this work. Functions were encoded in Python and MATLAB®. The Python file CRN_network_tools.py contains most of the scripts: the CRN2NET algorithm, the measures, the temporal evolution of the measures, and the visualization. The Python files in the null_model folder contain the scripts used to create the null model. All Python scripts developed for this work contain detailed information on the functions in the comments. The Python environment can be setup with CRN_network_tools_env.yml. The MATLAB® files (*.m in enzymatic oscillator.zip) were used separately to simulate the time series of the CRN. A README.txt is included as a user guide to setup the computational environment and how to execute the scripts. A Jupyter Notebook file (Complex Network analysis of oscillatory CRN.ipynb) is provided to allow for an interactive interface for importing, generating and manipulating Python functions and data.

### Script development

Details on the development of the CRN2NET algorithm and application of the network measures on the enzymatic oscillator are appended to ESI S2 and S3,† respectively. ESI S3† includes the implementation of the network measures, development of the random graph null model, and network measures applied on temporal behaviour.

## Data availability

The data that support the findings of this study are available in the ESI† of this article. Source codes for the simulated data can be downloaded from https://doi.org/10.4121/ac3c7c42-f367-41d7-bd3b-fa54714b3a1b. The readme file provides instructions to run the codes, and produce the full dataset used in this work.

## Author contributions

A. S. Y. W. and C. S. conceived and supervised the project. É. F. C. designed and T. P. revised step I of the procedure. Y. J. K. implemented the measures and the null model analysis (step 2 and step 3). É. F. C. implemented and D. V. K. designed the codes for the temporal analysis (step 4). A. S. Y. W., C. S., É. F. C. and Y. J. K. wrote the manuscript. É. F. C. prepared the extended materials. All authors contributed to revising the manuscript.

## Conflicts of interest

The authors declare no competing financial interests.

## Supplementary Material

SC-OLF-D4SC05234J-s001

SC-OLF-D4SC05234J-s002
