# Supplementary material for: Identify structures underlying out-of-equilibrium reaction networks with random graph analysis
Source: Chem Sci. 2025 Jan 8;16(7):3099–106. doi: 10.1039/d4sc05234j (PMC11736930; doi:10.1039/d4sc05234j)
Supplement: SC-016-D4SC05234J-s001 [file SC-016-D4SC05234J-s001.pdf]

Supplementary Information (SI) for Chemical Science.
This journal is © The Royal Society of Chemistry 2025

Supplementary Information

Identify structures underlying out-of-equilibrium reaction networks with random graph analysis

Éverton F. da Cunha,[1,2#] Yanna J. Kraakman,[3#] Dmitrii V. Kriukov,[1,2] Thomas van Poppel,[1] Clara Stegehuis,[3*] Albert S. Y. Wong[1,2*]

[1] Department of Molecules and Materials, Faculty of Science and Technology,

[2] BRAINS (Center for Brain-inspired Nano Systems),

[3] Department of Mathematical Operation Research, Faculty of Electrical Engineering, Mathematics and Computer Science,

University of Twente, Drienerlolaan 5, 7522 NH Enschede, the Netherlands

[#] authors contributed equally;

* Corresponding author: c.stegehuis@utwente.nl; albert.wong@utwente.nl

Content Page

CONTENT PAGE 2

S1. GENERAL 3

Methodology and Software 3

S2. DEVELOPMENT OF THE ALGORITHM CRN2NET 4

S2.1 Overview of the algorithm 4

S2.2 Build-up of the algorithm 4

S3. NETWORK MEASURES APPLIED ON THE ENZYMATIC OSCILLATOR 6

S3.1 Implementation of the network measures 6

S3.2 Development of the random graph null model 6

S3.2.1. Randomize reactions in a CRN 6

S3.2.2. Simulate randomized data 7

S3.3 Network measures applied on temporal behavior 8

S3.3.1. Simulating the temporal evolution of the trypsin oscillator based on the ODEs 8

S3.3.2. Simulating the temporal evolution of the species-species network G_2 8

S3.3.3. Simulating the temporal evolution of the network properties of the trypsin oscillator 9

NON-TEXTUAL ELEMENTS 10

S1. General

Methodology and Software

NetworkX (an open source Python package for complex networks) was used as the computational data structure to develop the species-species networks and implement the network measures used in this work. Functions were encoded in Python and MATLAB®. The Python file *CRN_network_tools.py* contains most of the scripts: the CRN2NET algorithm, the measures, the temporal evolution of the measures, and the visualization. The Python files in the *null_model* folder contain the scripts used to create the null model. All Python scripts developed for this work contain detailed information on the functions in the comments. The Python environment can be setup with *CRN_network_tools_env.yml*. The MATLAB® files (**.m* in *enzymatic oscillator.zip*) were used separately to simulate the time series of the CRN. A *README.txt* is included as a user guide to setup the computational environment and how to execute the scripts. A Jupyter Notebook file (*Complex Network analysis of oscillatory CRN.ipynb*) is provided to allow for an interactive interface for importing, generating and manipulating Python functions and data.

S2. Development of the algorithm CRN2NET

S2.1 Overview of the algorithm

The CRN2NET is the algorithm used to encode a chemical reaction network into a graph-based structure model, as explained in the main text. **Fig. S2** illustrates the overall procedure (step A to step F), but the algorithm starts from a list of chemical reactions that can be established by identifying the input and output elements for each reaction in the enzymatic reaction network, including the side reactions.

S2.2 Build-up of the algorithm

The outcome of the algorithm, the graph-based models G_1 or G_2 from main text **Fig. 3** – named species-species networks – are summarized as **Table S1** and **Table S2**. As an example, trypsin (Tr) autocatalysis comprises three elementary reactions. In **Step D** (Assign Species), classifies the reactants and products as output (*From*) and input (*To*) species respectively:

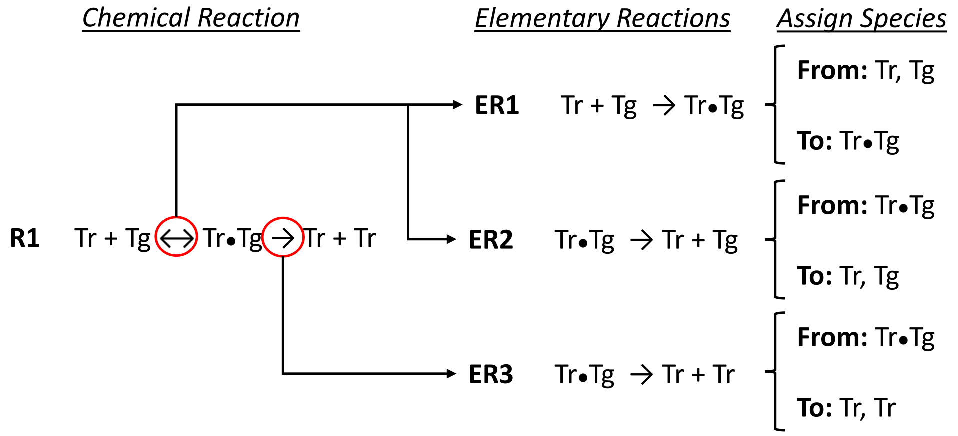

Next, Step **E** (Identify Direct Edges) creates the edges simply by connecting the identified input and output species with an arrow (*From* → *To*). Note that the arrow does not bear the meaning of a reaction (with an associated rate constant) but only indicates that there is a directed relation between the species. Therefore, multiple edges between two nodes are possible (known as edge multiplicity) even within the same elementary reaction, representing distinct relationships among the pair of species. Building on the same example from above, weights are defined according to the assigned species multiplicity as follows:

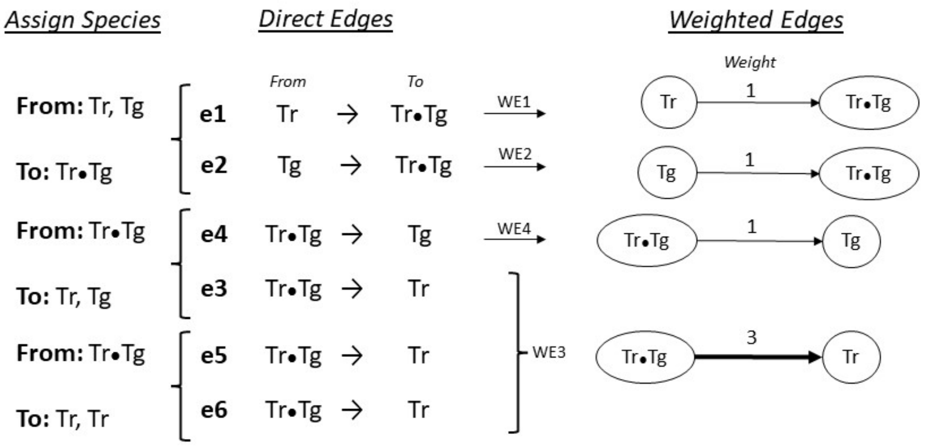

Directed edges e3, e5 and e6 are grouped together into one weighted edge, WE3, with a weight of three, represented by the thickness of the edge. **Fig. S3** depicts the species-species network constructed from the chemical species, as nodes, and their weighted directed edges. Overall, the outcome of the CRN2NET is a list of weighted direct edges (**Table S1**). The algorithm applied to the same system under out-of-equilibrium conditions yields **Table S2**. The list of chemical reactions for both system conditions (batch and flow) are included in the Extended Materials as *enzy_CRN_re_eqs_[batch/flow].txt*.

S3. Network measures applied on the enzymatic oscillator

S3.1 Implementation of the network measures

The network measures are derived from *NetworkX*. Specifically, when calculating the cluster coefficient, edge weights were not considered, and only feedback triangles were considered among the potential candidates of directed triangles. The algorithm can be found in the Extended Materials as **CRN network-tools.py**. Additionally, for betweenness centrality, weights were treated as multiplicities rather than the length of the path, as is done in the original *NetworkX* implementation. The three measures, introduced in the main text, were encoded in python and use the species-species network (**Table S1**, and **Table S2**) to produce **Table S3**.

S3.2 Development of the random graph null model

S3.2.1. Randomize reactions in a CRN

To create randomized network versions from our CRN, three steps are required:

1. Change the network from a weighted network to a multi-edge network, by replacing every edge of weight *w* by *w* copies of weight 1;
2. Uniformly randomize these edges by swapping pairs of edges;
3. Change the network back from a multi-edge network to a weighted network, by replacing all *w'* copies of an edge by one edge of weight *w'*.

The **Steps 1** and **3** are needed to randomize the edge weights. The total sum of the edge weights stays constant. The algorithm is given by the pseudocode below, where |E| is the size of the set of edges E, and can be found in *ConfigModel_EdgeSwapping_multidigraph.py*. Note that the algorithm never changes the degrees of the nodes. The acceptance probability that is used, makes sure that there is no bias towards creating networks with edges with small weight. In particular, it ensures that the probability of creating any network with these degrees is approximately equal.

```
let G be the network, with edges given by the set E
for every edge e in E:
    let w = weight(e)
    replace e in E by w edges of weight 1
repeat 50 * |E| times:
    pick two edges (a,b), (c,d) uniformly at random from E
    let (a,d), (c,b) be the shuffled edges
    let r be a random number between 0 and 1
    if r > 1/(multiplicity((a,b)) * multiplicity((c,d)):
        accept shuffle, so remove edges (a,b),(c,d) from E, add edges (a,d),(c,b) to E
    else:
        reject shuffle, so do not change anything.
for every edge e in E:
    let w’ = multiplicity(e)
    replace all w’ copies of e in E by one edge with weight w’
return G, E
```

S3.2.2. Simulate randomized data

Generating the data for the random null model requires two steps:

1. Create 10,000 random network samples and compute the *cc* and *bc* values for every node in every random network;
2. For every node, show the null model data in a boxplot, together with the original data point.

The algorithm is given by the pseudocode below, and can be found in the Extended Materials as *Create_null_model_data.py*.

```
let G be a network, with nodes given by the set V
for every node v in V:
    create an empty array cc_v
    create an empty array bc_v
repeat 10,000 times:
    create a random network G' using the previous algorithm
    for every node v in V:
        compute the cc value of v in G' and append the value to cc_v
        compute the bc value of v in G' and append the value to bc_v
 for every node v in V:
    create a boxplot from the values in cc_v
    add the cc value of v in G to this boxplot as the original data value
    create a boxplot form the values in bc_v
    add the bc value of v in G to this boxplot as the original data value
```

S3.3 Network measures applied on temporal behavior

S3.3.1. Simulating the temporal evolution of the trypsin oscillator based on the ODEs

Three MATLAB® scripts were developed and used to simulate the time series. Specifically, *ode_trypsin_osc.m* encodes the ODEs functions of the CRN system; *masterscript_ode_param.m* solves the ODEs with the necessary conditions; and *data_mining_master.m* runs the simulation and saves the produced timeseries. These scripts are adapted based on existing codes published earlier[9] and used to simulate the behavior of the oscillator, depicted as concentration over time. Simulations for main text **Fig. 5a** were performed with t=100 h and with the initial conditions: V=250 µl, $[Tg]_0$=200 µM, $[Tr]_0$=2 µM, $[Pro\text{-}I]_0$=1.5 mM, Ap=30 U/ml.

S3.3.2. Simulating the temporal evolution of the species-species network G_2

The simulated time series were converted into binary values to identify the temporal evolution of G_2. We consider that an edge can only exist when the associated node is present. For each time point, we calculated the second derivative of the species concentration to determine the threshold value for the individual node. In particular, we say that the temporal threshold for when a species

becomes present or absent is when this second derivative equals zero. This dynamic threshold is able to adapt to changes on the species level, as a the absolute concentrations of the individual species differ significantly (enzymes are in the micromolar range, while small molecules are in the millimolar range). Then, we converted the original set of time series (with concentrations as units) into a set of modified time series with binary values (with '1' and '0' assigned to the values above and below the threshold, respectively). Subsequently, the binary time series determine when the edges in G_2 are present. Specifically, we only say that edges are present when they direct from a node that is present (value 1), while edges that direct from a node with value 0 are deleted at that time step. The Python function *t_snap* takes each time point of the binary time series, connects the nodes that were assigned as 1 with an edges, as in **Table S2**, and produces a species-species network for that specific system moment. Repeating this procedure for every time point during a single oscillation yields a sequence of snapshots, which we animated with a Graphics Interchange Format, gif, in **Fig. S4**.

S3.3.3. Simulating the temporal evolution of the network properties of the trypsin oscillator

We applied our implementation of clustering coefficient and betweenness centrality to examine the temporal evolution of the network properties of the enzymatic oscillator. For each flow rate value, cc_5 and bc_7 is measured for the full concentration time, 180 h. Repeating this procedure for a flow rate in the range of 0-500 μL h^{-1}, with an increment of 1 μL h^{-1}, yields a sequence of time series snapshots of the evolution of the *Tr* concentration and network measures, which are represented as gif in **Fig. S5**. As can be expected, the desired dynamic behavior (sustained oscillations) were only found between a restricted range of flow rates, from 5 μL h^{-1} to 65 μL h^{-1}. The temporal evolution of *cc* and *bc* follows the patterns of the oscillation, even when they are not sustained.

Non-Textual Elements

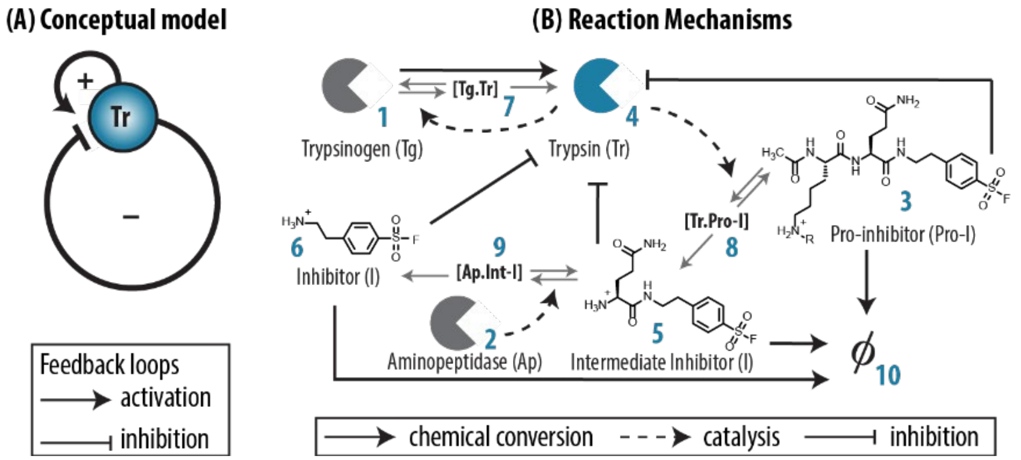

Fig. S1. A previously developed trypsin (*Tr*) network, depicted as a conceptual model (A) comprising feedback loops. (B) The reaction scheme with the underlying reaction mechanisms used for developing the script 'CRN2NET'.

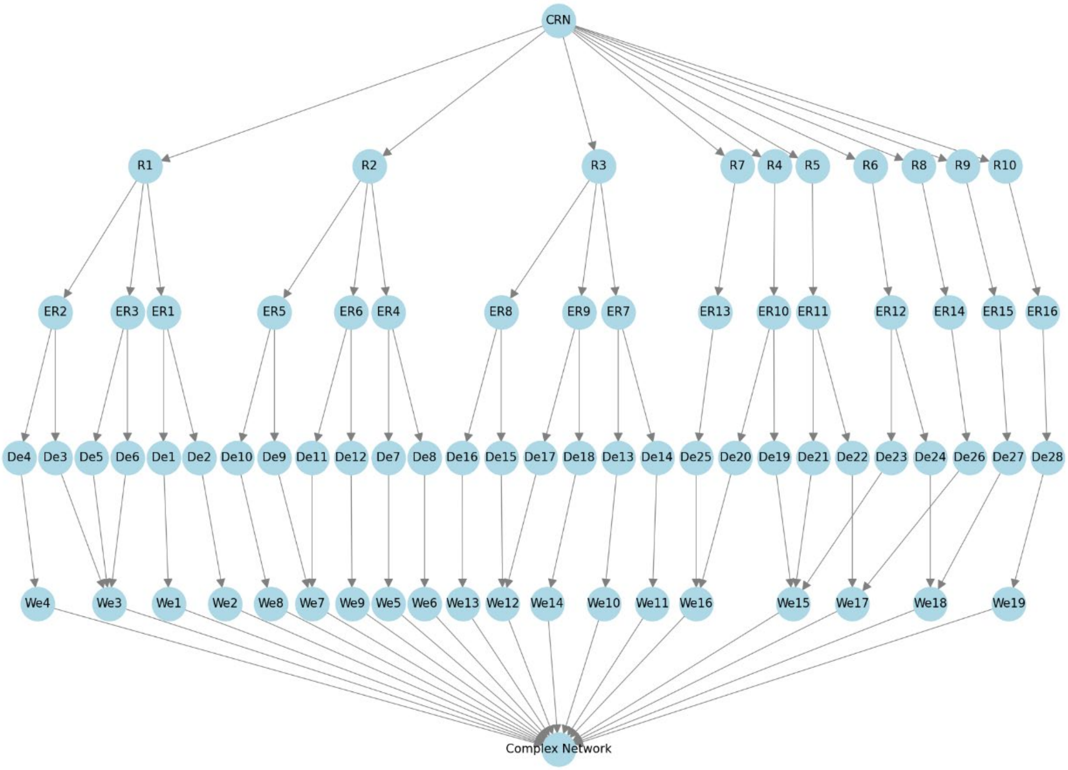

Fig. S2. Overview of the process involved in encoding the enzymatic reaction network into a species-species network (or, more generally, a complex network). 'R#' denotes the chemical reactions (with '#' ranging from 1 to 10). 'ER#' denotes the elementary reactions (with '#' ranging from 1 to 16). 'De#' denotes the directed edges (with '#' ranging from 1 to 28). 'We#' denotes the weighted edges (with '#' ranging from 0 to 19).

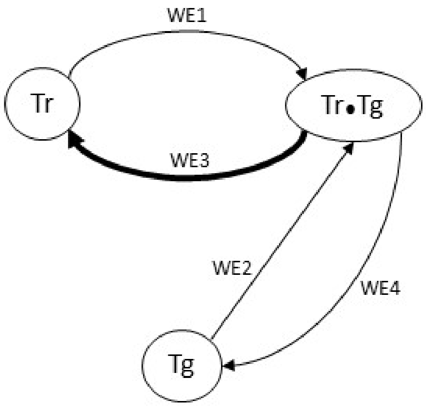

Fig. S3. Example of a species-species network. The CRN2NET applied on reaction R1 (**Table S1**) yields the species-species network representing the autocatalytic conversion of *Tg* into *Tr*. The weight of the edges is visualized by the thickness of the arrow.

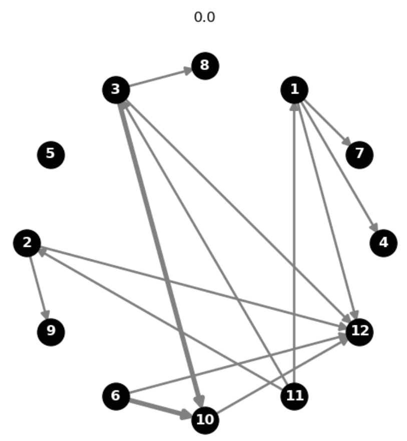

Fig. S4. Temporal evolution of the species-species network G_2 during a single oscillation. The fraction of a period is indicated on the top of the graph. The animated image sequence is provided in the Extended Materials: *SupplementaryInformation_FigS3.gif.*

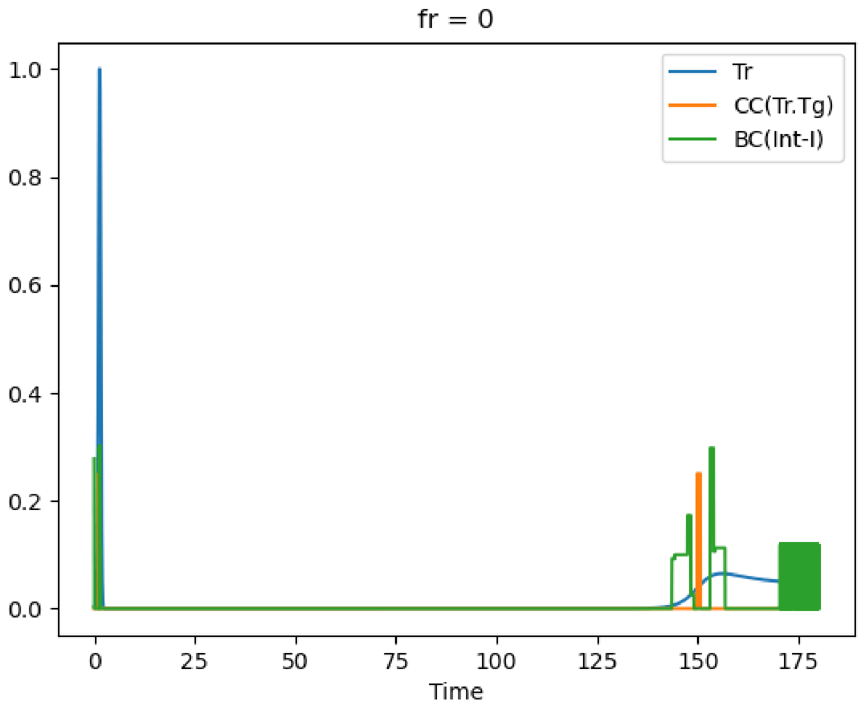

Fig. S5. Temporal evolution of the oscillator and the network measures as a function of flow. The dynamic behavior, depicted as concentration of *Tr* over time, and the structural behavior, depicted as network measures cc_5 and bc_7 over time. The flow rate (fr, in µL h^{-1})—reciprocal of space velocity—is indicated on the top of the graph. The animated image sequence is provided in the Extended Materials: *SupplementaryInformation_FigS4.gif*.

Table S1. Summary of the nodes and edges associated with the Species-species network under equilibrium conditions, G_1.

A: CM	B: Specify Reaction Mechanisms		C: Split into Elementary Steps	D: Assign Species		E: Identify Directed Edges
	Process description	*Reaction Mechanisms*		*From*	*To*	
**POSITIVE FEEDBACK**	Trypsin **autocatalysis**	**R1** Tr + Tg ↔ Tr·Tg → Tr + Tr	**ER1** Tr + Tg → Tr·Tg	Tr	Tr·Tg	**De1** Tr → Tr·Tg
				Tg	Tr·Tg	**De2** Tg → Tr·Tg
			**ER2** Tr·Tg → Tr + Tg	Tr·Tg	Tr	**De3** Tr·Tg → Tr
				Tr·Tg	Tg	**De4** Tr·Tg → Tg
			**ER3** Tr·Tg → Tr + Tr	Tr·Tg	Tr	**De5** Tr·Tg → Tr
				Tr·Tg	Tr	**De6** Tr·Tg → Tr
**NEGATIVE FEEDBACK**	Pro-inhibitor **activation**	**R2** Tr + Pro-I ↔ Tr·Pro-I → Tr + Int-I	**ER4** Tr + Pro-I → Tr·Pro-I	Tr	Tr·Pro-I	**De7** Tr → Tr·Pro-I
				Pro-I	Tr·Pro-I	**De8** Pro-I → Tr·Pro-I
			**ER5** Tr·Pro-I → Tr + Pro-I	Tr·Pro-I	Tr	**De9** Tr·Pro-I → Tr
				Tr·Pro-I	Pro-I	**De10** Tr·Pro-I → Pro-I
			**ER6** Tr·Pro-I → Tr + Int-I	Tr·Pro-I	Tr	**De11** Tr·Pro-I → Tr
				Tr·Pro-I	Int-I	**De12** Tr·Pro-I → Int-I
	**Delay**ed inhibitor activation	**R3** Ap + Int-I ↔ Ap·Int-I → Ap + I	**ER7** Ap + Int-I → Ap·Int-I	Ap	Ap·Int-I	**De13** Ap → Ap·Int-I
				Int-I	Ap·Int-I	**De14** Int-I → Ap·Int-I
			**ER8** Ap·Int-I → Ap + Int-I	Ap·Int-I	Ap	**De15** Ap·Int-I → Ap
				Ap·Int-I	Int-I	**De16** Ap·Int-I → Int-I
			**ER9** Ap·Int-I → Ap + I	Ap·Int-I	Ap	**De17** Ap·Int-I → Ap
				Ap·Int-I	I	**De18** Ap·Int-I → I
	Trypsin **inhibition** by active inhibitor	**R4** Tr + I → P	**ER10** Tr + I → P	Tr	P	**De19** Tr → P
				I	P	**De20** I → P
**SIDE REACTIONS**	Trypsin inhibition by intermediate inhibitor	**R5** Tr + Int-I → P	**ER11** Tr + Int-I → P	Tr	P	**De21** Tr → P
				Int-I	P	**De22** Int-I → P
	Trypsin inhibition by pro inhibitor	**R6** Tr + Pro-I → P	**ER12** Tr + Pro-I → P	Tr	P	**De23** Tr → P
				Pro-I	P	**De24** Pro-I → P
	Hydrolysis of active inhibitor	**R7** I → P	**ER13** I → P	I	P	**De25** I → P
	Hydrolysis of intermediate inhibitor	**R8** Int-I → P	**ER14** Int-I → P	Int-I	P	**De26** Int-I → P
	Hydrolysis of pro inhibitor	**R9** Pro-I → P	**ER15** Pro-I → P	Pro-I	P	**De27** Pro-I → P
	Trypsinogen auto-activation	**R10** Tg → Tr	**ER16** Tg → Tr	Tg	Tr	**De28** Tg → Tr

F: Define Weighted Edges		
	Edges	*Weights*
**We1**	Tr → Tr·Tg	**1**
**We2**	Tg → Tr·Tg	**1**
**We3**	Tr·Tg → Tr	**3**
**We4**	Tr·Tg → Tg	**1**
**We5**	Tr → Tr·Pro-I	**1**
**We6**	Pro-I → Tr·Pro-I	**1**
**We7**	Tr·Pro-I → Tr	**2**
**We8**	Tr·Pro-I → Pro-I	**1**
**We9**	Tr·Pro-I → Int-I	**1**
**We10**	Ap → Ap·Int-I	**1**
**We11**	Int-I → Ap·Int-I	**1**
**We12**	Ap·Int-I → Ap	**2**
**We13**	Ap·Int-I → Int-I	**1**
**We14**	Ap·Int-I → I	**1**
**We15**	Tr → P *	**3**
**We16**	I → P *	**2**
**We17**	Int-I → P	**2**
**We18**	Pro-I → P	**2**
**We19**	Tg → Tr	**1**

Species-Species Network	
**Nodes**	**10**
**Edges (Weighted edges)**	**28 (19)**

CM abbreviates conceptual model. *Weighted edges that comprise processes for the negative feedback and side reactions as defined in the original model.

Table S2. Summary of the nodes and edges associated with the Species-species network under out-of-equilibrium conditions, G_2.

A: CM	B: Specify Reaction Mechanisms		C: Split into Elementary Steps	D: Assign Species		E:Identify Directed Edges
	Process description	*Reaction Mechanisms*		*From*	*To*	
**POSITIVE FEEDBACK**	Trypsin **autocatalysis**	**R1** Tr + Tg ↔ Tr·Tg → Tr + Tr	**ER1** Tr + Tg → Tr·Tg	Tr	Tr·Tg	**De1** Tr → Tr·Tg
				Tg	Tr·Tg	**De2** Tg → Tr·Tg
			**ER2** Tr·Tg → Tr + Tg	Tr·Tg	Tr	**De3** Tr·Tg → Tr
				Tr·Tg	Tg	**De4** Tr·Tg → Tg
			**ER3** Tr·Tg → Tr + Tr	Tr·Tg	Tr	**De5** Tr·Tg → Tr
				Tr·Tg	Tr	**De6** Tr·Tg → Tr
**NEGATIVE FEEDBACK**	Pro-inhibitor **activation**	**R2** Tr + Pro-I ↔ Tr·Pro-I → Tr + Int-I	**ER4** Tr + Pro-I → Tr·Pro-I	Tr	Tr·Pro-I	**De7** Tr → Tr·Pro-I
				Pro-I	Tr·Pro-I	**De8** Pro-I → Tr·Pro-I
			**ER5** Tr·Pro-I → Tr + Pro-I	Tr·Pro-I	Tr	**De9** Tr·Pro-I → Tr
				Tr·Pro-I	Pro-I	**De10** Tr·Pro-I → Pro-I
			**ER6** Tr·Pro-I → Tr + Int-I	Tr·Pro-I	Tr	**De11** Tr·Pro-I → Tr
				Tr·Pro-I	Int-I	**De12** Tr·Pro-I → Int-I
	**Delay**ed inhibitor activation	**R3** Ap + Int-I ↔ Ap·Int-I → Ap + I	**ER7** Ap + Int-I → Ap·Int-I	Ap	Ap·Int-I	**De13** Ap → Ap·Int-I
				Int-I	Ap·Int-I	**De14** Int-I → Ap·Int-I
			**ER8** Ap·Int-I → Ap + Int-I	Ap·Int-I	Ap	**De15** Ap·Int-I → Ap
				Ap·Int-I	Int-I	**De16** Ap·Int-I → Int-I
			**ER9** Ap·Int-I → Ap + I	Ap·Int-I	Ap	**De17** Ap·Int-I → Ap
				Ap·Int-I	I	**De18** Ap·Int-I → I
	Trypsin **inhibition** by active inhibitor	**R4** Tr + I → P	**ER10** Tr + I → P	Tr	P	**De19** Tr → P
				I	P	**De20** I → P
**SIDE REACTIONS**	Trypsin inhibition by intermediate inhibitor	**R5** Tr + Int-I → P	**ER11** Tr + Int-I → P	Tr	P	**De21** Tr → P
				Int-I	P	**De22** Int-I → P
	Trypsin inhibition by pro inhibitor	**R6** Tr + Pro-I → P	**ER12** Tr + Pro-I → P	Tr	P	**De23** Tr → P
				Pro-I	P	**De24** Pro-I → P
	Hydrolysis of active inhibitor	**R7** I → P	**ER13** I → P	I	P	**De25** I → P
	Hydrolysis of intermediate inhibitor	**R8** Int-I → P	**ER14** Int-I → P	Int-I	P	**De26** Int-I → P
	Hydrolysis of pro inhibitor	**R9** Pro-I → P	**ER15** Pro-I → P	Pro-I	P	**De27** Pro-I → P
	Trypsinogen auto-activation	**R10** Tg → Tr	**ER16** Tg → Tr	Tg	Tr	**De28** Tg → Tr
**INFLOW**	In(Tg)	**R11** S → Tg	**ER17** S → Tg	S	Tg	**De29** S → Tg
	In(Pro-I)	**R12** S → Pro-I	**ER18** S → Pro-I	S	Pro-I	**De30** S → Pro-I
	In(Ap)	**R13** S → Ap	**ER19** S → Ap	S	Ap	**De31** S → Ap
**OUTFLOW**	Out(Tg)	**R14** Tg → W	**ER20** Tg → W	Tg	W	**De32** Tg → W
	Out(Tr)	**R15** Tr → W	**ER21** Tr → W	Tr	W	**De33** Tr → W
	Out(Tr·Tg)	**R16** Tr·Tg → W	**ER22** Tr·Tg → W	Tr·Tg	W	**De34** Tr·Tg → W
	Out(Pro-I)	**R17** Pro-I → W	**ER23** Pro-I → W	Pro-I	W	**De35** Pro-I → W
	Out(Tr·Pro-I)	**R18** Tr·Pro-I → W	**ER24** Tr·Pro-I → W	Tr·Pro-I	W	**De36** Tr·Pro-I → W
	Out(Int-I)	**R19** Int-I → W	**ER25** Int-I → W	Int-I	W	**De37** Int-I → W
	Out(Ap)	**R20** Ap → W	**ER26** Ap → W	Ap	W	**De38** Ap → W
	Out(Ap·Int-I)	**R21** Ap·Int-I → W	**ER27** Ap·Int-I → W	Ap·Int-I	W	**De39** Ap·Int-I → W
	Out(I)	**R22** I → W	**ER28** I → W	I	W	**De40** I → W
	Out(P)	**R23** P → W	**ER29** P → W	P	W	**De41** P → W

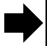

F: Define Weighted Edges		
	Edges	*Weights*
**We1**	Tr → Tr·Tg	**1**
**We2**	Tg → Tr·Tg	**1**
**We3**	Tr·Tg → Tr	**3**
**We4**	Tr·Tg → Tg	**1**
**We5**	Tr → Tr·Pro-I	**1**
**We6**	Pro-I → Tr·Pro-I	**1**
**We7**	Tr·Pro-I → Tr	**2**
**We8**	Tr·Pro-I → Pro-I	**1**
**We9**	Tr·Pro-I → Int-I	**1**
**We10**	Ap → Ap·Int-I	**1**
**We11**	Int-I → Ap·Int-I	**1**
**We12**	Ap·Int-I → Ap	**2**
**We13**	Ap·Int-I → Int-I	**1**
**We14**	Ap·Int-I → I	**1**
**We15**	Tr → P *	**3**
**We16**	I → P *	**2**
**We17**	Int-I → P	**2**
**We18**	Pro-I → P	**2**
**We19**	Tg → Tr	**1**
**We20**	S → Tg	**1**
**We21**	S → Pro-I	**1**
**We22**	S → Ap	**1**
**We23**	Tg → W	**1**
**We24**	Tr → W	**1**
**We25**	Tr·Tg → W	**1**
**We26**	Pro-I → W	**1**
**We27**	Tr·Pro-I → W	**1**
**We28**	Int-I → W	**1**
**We29**	Ap → W	**1**
**We30**	Ap·Int-I → W	**1**
**We31**	I → W	**1**
**We32**	P → W	**1**

Species-Species Network	
**Nodes**	**12**
**Edges (Weighted edges)**	**43 (32)**

CM abbreviates conceptual model. *Weighted edges that comprise processes for the negative feedback and side reactions as defined in the original model.

Table S3: Network measured applied on the species-species networks G_1 and G_2.

Node		Degree		*cc*		*bc*	
#	*Species*	*G_1*	*G_2*	*G_1*	*G_2*	*G_1*	*G_2*
**1**	Tg	3	5	1	0.2	0	0.0365
**2**	Ap	3	5	0	0	0	0.0429
**3**	Pro-I	4	6	0	0	0.0044	0.0474
**4**	Tr	11	12	0.1429	0.1	0.4133	0.2906
**5**	Int-I	5	6	0	0	0.3447	0.2455
**6**	I	3	4	0	0	0.0204	0.0147
**7**	Tr.Tg	6	7	0.5	0.25	0.0667	0.2906
**8**	Tr.Pro-I	6	7	0	0	0.4889	0.3516
**9**	Ap.Int-I	6	7	0	0	0.3191	0.25
**10**	P	9	10	0	0	0	0
**11**	S	-	3	-	0	-	0
**12**	W	-	10	-	0	-	0
