# Supplementary figures and images for: Identify structures underlying out-of-equilibrium reaction networks with random graph analysis
Source: Chem Sci. 2025 Jan 8;16(7):3099–106. doi: 10.1039/d4sc05234j (PMC11736930; doi:10.1039/d4sc05234j)

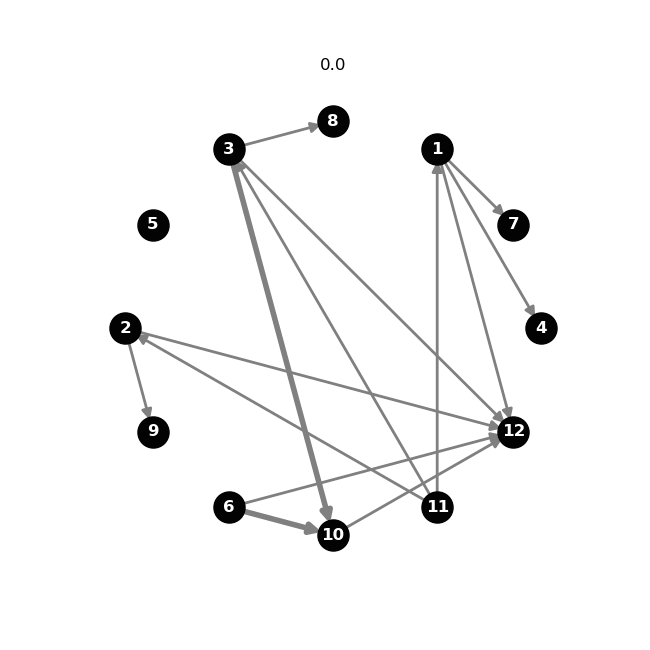

Supplement: SC-016-D4SC05234J-s002 [file SC-016-D4SC05234J-s002.zip › ExtendedMaterials/SupplementaryInformation_FigS3.gif]

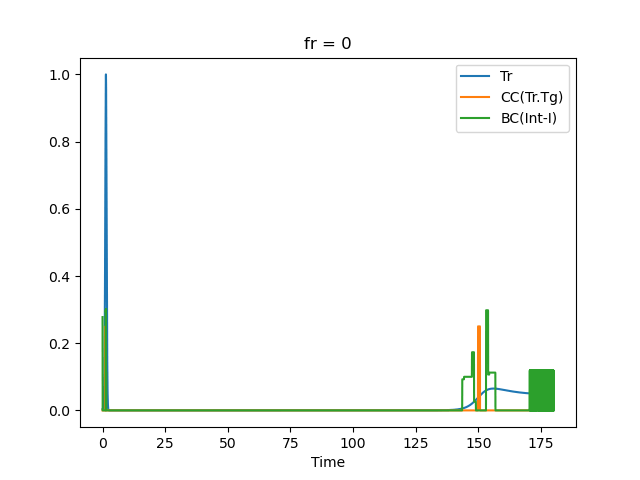

Supplement: SC-016-D4SC05234J-s002 [file SC-016-D4SC05234J-s002.zip › ExtendedMaterials/SupplementaryInformation_FigS4.gif]
